# Dynamic fluctuations in ascending heart-to-brain communication under mental stress

**DOI:** 10.1152/ajpregu.00251.2022

**Published:** 2023-02-20

**Authors:** Diego Candia-Rivera, Kian Norouzi, Thomas Zoëga Ramsøy, Gaetano Valenza

**Affiliations:** ^1^Department of Information Engineering & Bioengineering and Robotics Research Center E. Piaggio, School of Engineering, University of Pisa, Pisa, Italy; ^2^Department of Applied Neuroscience, Neurons, Inc., Taastrup, Denmark; ^3^Faculty of Management, University of Tehran, Tehran, Iran; ^4^Faculty of Neuroscience, Singularity University, Santa Clara, California, United States

**Keywords:** brain-heart interplay, mental stress, physiological modeling, sympathovagal control

## Abstract

Dynamical information exchange between central and autonomic nervous systems, as referred to functional brain-heart interplay, occurs during emotional and physical arousal. It is well documented that physical and mental stress lead to sympathetic activation. Nevertheless, the role of autonomic inputs in nervous system-wise communication under mental stress is yet unknown. In this study, we estimated the causal and bidirectional neural modulations between electroencephalogram (EEG) oscillations and peripheral sympathetic and parasympathetic activities using a recently proposed computational framework for a functional brain-heart interplay assessment, namely the sympathovagal synthetic data generation model. Mental stress was elicited in 37 healthy volunteers by increasing their cognitive demands throughout three tasks associated with increased stress levels. Stress elicitation induced an increased variability in sympathovagal markers, as well as increased variability in the directional brain-heart interplay. The observed heart-to-brain interplay was primarily from sympathetic activity targeting a wide range of EEG oscillations, whereas variability in the efferent direction seemed mainly related to EEG oscillations in the γ band. These findings extend current knowledge on stress physiology, which mainly referred to top-down neural dynamics. Our results suggest that mental stress may not cause an increase in sympathetic activity exclusively as it initiates a dynamic fluctuation within brain-body networks including bidirectional interactions at a brain-heart level. We conclude that directional brain-heart interplay measurements may provide suitable biomarkers for a quantitative stress assessment and bodily feedback may modulate the perceived stress caused by increased cognitive demand.

## Graphical abstract

**Figure:**
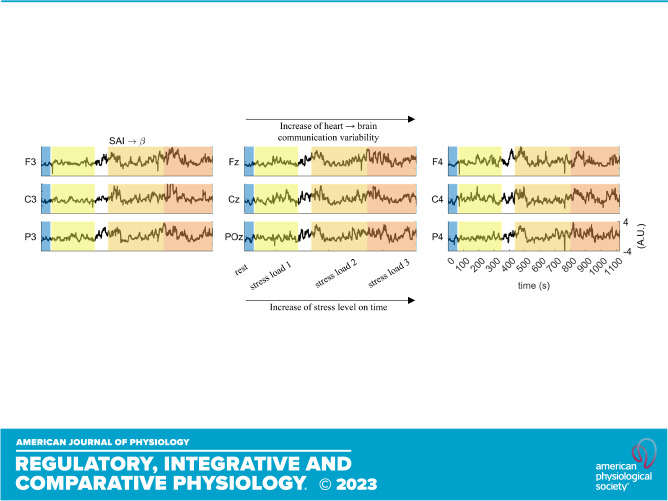

## INTRODUCTION

Human physiology entails constant and dynamic adaptations in response to cognitive demand through regulatory mechanisms. As part of the regulatory processes, monitoring of peripheral bodily activity contributes to the adaptation to changes in the self or the environment (1). To illustrate, these processes may stimulate specific behaviors that allow finding shelter or food in extreme conditions. Although such physiological adjustments comprehensively refer to “homeostasis,” adjustments that anticipate future needs refer to allostasis (2). Allostasis thus requires cognitive functions, such as subjective perception, understanding, learning, and memorizing (2).

From a holistic point of view, the physiological responses to cognitive load refer to “mental stress,” which can be elicited by memory, arithmetic, and increased cognitive demand tasks (3). Physical stress involves the physiological responses triggered by homeostatic regulations to bodily conditions, emerging from physical exercise or environmental changes (e.g., temperature or atmospheric pressure) (3). Mental and physical stress encompasses physiological responses from different brain structures, together with responses from peripheral systems (4). The neurophysiology of stress sets the hypothalamus as a central component, in which the paraventricular nucleus is the main integrator of stressors, activating systems such as the sympathetic-adreno-medullar and hypothalamus-pituitary-adrenal axes (5). The brain structures actively involved in stress responses include the prefrontal cortex (6) and the amygdala, whose activity is also associated with emotional processing (7). Prefrontal projections to the amygdala (8), as well as hippocampus projections to the amygdala and prefrontal cortex (9) are involved as well. Underlying stress mechanisms have also been captured in electroencephalogram (EEG) studies, showing a high diversity of responses, including hemispheric changes in α power and wide-range variability in the EEG spectrum (10, 11).

The central autonomic network integrates the interoceptive and exteroceptive information to promote physiological and behavioral changes that allow adaptation to ongoing challenges, including stress conditions (4, 12–14). Previous studies highlighted a close relationship between stress and sympathetic nervous system activity (15–17), which has also been assessed through series of heart rate variability (18–22), skin conductance (22, 23), breathing (24), body temperature (25, 26), and blood pressure (18, 19, 22). Gastrointestinal (27, 28), endocrine (29), and immune responses (30) were also taken into account to investigate the functional link between stress and sympathetic response. On the other hand, acute stress triggers concurrent fluctuations in heart rate variability and functional connectivity between the central executive and default mode networks (31). Neural responses to heartbeats have been described as a potential indicator of stress because of the correlations found with sympathetic indexes (22). Similarly with the correlations found between EEG power and autonomic indexes under mental stress (32).

Since stress conditions may induce emotional responses (33), physiological responses to stress (i.e., stress regulation) may be linked to physiological mechanisms of emotion regulation (34). Indeed, although cardiovascular dynamics are modulated by emotional processing (35, 36), modulation activity of the functional brain-heart interactions have been observed under thermal stress and thermoregulatory responses (26, 37), as well as emotional processing (38). Accordingly, cardiac interoceptive feedback seems actively involved under stressful conditions (39, 40), and a wider involvement of the functional brain-peripheral body axis in mental stress has already been hypothesized (41). Nonetheless, the functional brain-peripheral body physiology associated with mental stress is yet unknown. We have hypothesized that the embodiment of mental stress is reflected in bidirectional brain-heart interplay, with specific involvement of sympathetic and vagal dynamics. Accordingly, this study aims to uncover the directional brain-heart interplay mechanisms involved in mental stress induced through visual stimulation and memory tasks. Specifically, we exploited our recently proposed sympathovagal synthetic data generation model (SV-SDG) (37) to uncover the mutual functional communication between cortical oscillations, as measured through EEG, and cardiac sympathetic/parasympathetic activities, estimated from heartbeat dynamics. The SV-SDG model provides time-varying estimates of the causal interplay between sympathetic/parasympathetic activities and EEG oscillations in a specific frequency band. The framework embeds a heartbeat generation model based on the estimation of sympathetic and parasympathetic activities from Laguerre expansions of the heartbeat series (42).

## MATERIALS AND METHODS

### Data Set Description

Data were gathered from 37 healthy participants (age median 30 yr, age range 22–45 yr, 20 males, 17 females) who underwent mental stress elicitation tasks. Participants were asked to sit comfortably and follow instructions on a screen. Recordings of physiological signals included EEG (9-channel, Biopac B-Alert) and one lead ECG, both sampled at 256 Hz.

This study was performed at Neurons, Inc., Taastrup, Denmark, in accordance with the Declaration of Helsinki and followed the rules and laws of the Danish Data Protection Agency. Data protection policy also followed the European Union law of the General Data Protection Regulation, as well as the ethical regulations imposed by the Neuromarketing Science and Business Association, Article 6. The participants signed a written informed consent to participate in this study. Each participant’s biometric data, survey responses, and other types of data were anonymized and only contained the log number as the unique identifier. Personal information cannot be identified from the log number. The data processing was approved by the ethics committee “Comitato Bioetico di Ateneo” of the University of Pisa.

### Experimental Protocol

Stress induction was performed through a parametric modulation protocol (43, 44) with increased stress levels over time. The protocol comprised four stressing conditions, including 1 min rest and three different stress load tasks, each of which lasted 15 min approximately (5 min each task). The stressors were presented in the same order to all participants. The first stress load condition consisted in watching a documentary. The second stress load condition consisted in watching a documentary concurrently with performing a digit span task. The third stress load condition consisted in watching a documentary and performing the digit span task and the red box task. For each condition, participants were asked to self-assess and report the perceived stress level through a discrete scale from 1 to 7 (from low- to high-stress score).

**Figure 1.**
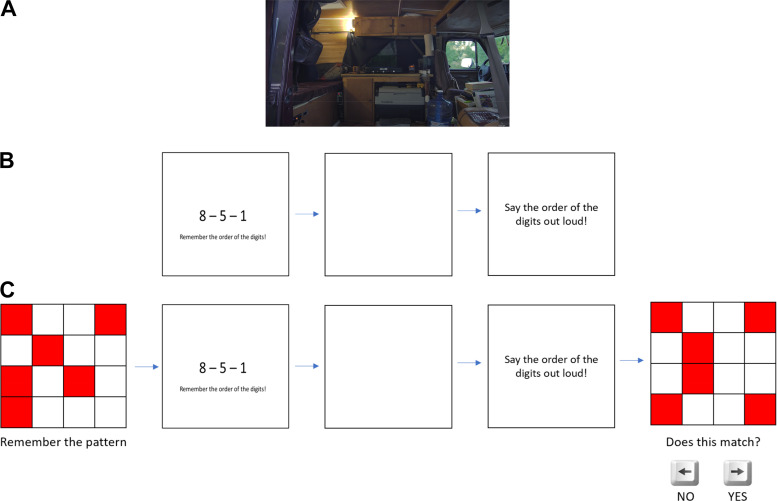
Exemplary stress elicitation images. *A*: sample image from stress load *condition 1*. The displayed video is an excerpt from the documentary “*The Reality of Van Life*” (Different Media 2018). The video was presented for 5 min. *B*: sample image from stress load *condition 2*. The experimental condition consisted in watching the documentary from (*A*) simultaneously to performing a digit span task of memorizing 3-digit sequences, for approximately 5 min. *C*: sample figure from stress load *condition 3*. The experimental condition consisted in watching the documentary from (*A*), performing the digit span task from (*B*), and performing the red box task of memorizing 4 × 4 patterns, for approximately 5 min.

More specifically, the first 5 min of the documentary “*The Reality of Van Life*”, Different Media © 2018, was projected onto a screen as the first stressor (Fig. 1*A*). The digit span task started with a fixation cross for 1.5 s. Then, three digits were presented for 5 s, followed by a blank screen for 4 s. The participant was then asked to verbally state the three digits in up to 5 s (Fig. 1*B*). The red box task, which ran in parallel to the digit span task (Fig. 1*C*), started with a fixation cross for 1.5 s. Then a red box (4 × 4 red and white box pattern) was presented for 3 s. Next, the three digits were presented for 5 s, followed by a blank screen for 4 s. Then the participant was asked to verbally state the three digits in up to 5 s. Consecutively, a red box was presented, and the participant was asked if the pattern matches the previously presented one (yes or no answer).

### EEG Preprocessing

EEG data were preprocessed using MATLAB R2022a and Fieldtrip Toolbox (45). EEG data were bandpass filtered with a Butterworth filter of order 4, between 0.5 and 45 Hz. Large movement artifacts were visually identified and removed manually from independent component space and wavelet filtering. Consecutively, the Independent Component Analysis (ICA) was computed to visually recognize and reject the eye movements and cardiac-field artifacts from the EEG data. One lead ECG was included as an additional input to the ICA to enhance the process of finding cardiac artifacts. Once the ICA components with eye movements and cardiac artifacts were visually identified, they were removed to reconstruct the EEG series. Channels were rereferenced using a common average, which is the most appropriate for brain-heart interplay estimations (46).

The EEG spectrogram was computed using the short-time Fourier transform with a Hanning taper. Calculations were performed through a sliding time window of 2 s with 50% overlap, resulting in a spectrogram resolution of 1 s and 0.5 Hz. Then, time series were integrated within five frequency bands (δ: 1–4 Hz, θ: 4–8 Hz, α: 8–12 Hz, β: 12–30 Hz, γ: 30–45 Hz).

### ECG Data Processing

ECG time series were bandpass filtered using a Butterworth filter of order 4, between 0.5 and 45 Hz. The R peaks from the QRS waves were detected in a procedure based on the template-matching method (46). All the detected peaks were visually inspected over the original ECG, along with the interbeat intervals histogram. Manual corrections were performed where needed and guided from the automatic detection of ectopic beats (47).

### Estimation of Sympathetic and Parasympathetic Activities

Heart rate variability (HRV) series are usually analyzed by using the Fourier transform, which represents HRV in the frequency domain. This method groups the values into different frequency ranges [very low frequency (VLF): < 0.04 Hz, low frequency (LF): 0.04–0.15 Hz, and high frequency (HF): 0.15–0.4 Hz]. However, an alternative approach is the use of autoregressive models, which have the advantage of reducing the dimensionality of the frequency space by defining a limited number of preferred oscillations. This method has been widely used in autonomic assessment, as the frequencies within the HF range can be directly associated with vagal dynamics (48). However, there are limitations to this method, such as the fact that the LF range contains both vagal and sympathetic dynamics (49, 50). To overcome these limitations, we recently proposed the sympathetic and parasympathetic activity indices (SAI and PAI, respectively), which use Laguerre functions as an alternative way to analyze HRV through autoregressive models. These functions are characterized by a specific “order” and “α” value, which can vary from zero to any positive integer. Indices of sympathetic and parasympathetic activities are derived from characterizing and predicting each heartbeat event by using a combination of past information of RR intervals (R-to-R peak intervals). This approach improves the identification of model parameters needed to estimate the cardiac sympathetic and parasympathetic activities (48).

Methodologically, the series of RR intervals were convolved with a set of Laguerre functions φ_j_, as shown in *Eq. 1*:

(*1*) $$L_{j}\left(k\right)=\sum_{n\;=\;0}^{k-1}\varphi_{j}\left(n\right)\cdot \text{RR}\left(k-n-1\right).$$

Therefore, the RR series can be expanded using the convolved Laguerre functions *L*(*k*) = [*L*_0_(*k*),*L*_1_(*k*),…,*L*_8_(*k*)]*^T^*, and the theoretical autoregressive model can be used to separate the sympathetic and parasympathetic components as follows:

(*2*) $$\text{RR}(k)\;=\underset{\text{baseline}}{\underset{︸}{g_{0}(k)}}+\;\underset{\text{sympathetic}}{\underset{︸}{\sum_{j\;=\;0}^{1}g_{1,\;j}(k)\;\cdot \;L_{j}(k)}}+\underset{\text{parasympathetic}}{\underset{︸}{\sum_{j\;=\;2}^{8}g_{1,\;j}\left(k\right)L_{j}\left(k\right)}}.$$

The time-varying Laguerre coefficients *g*(*k*) = [*_g_*_0_(*k*),*g*_1,0_(*k*),…,*g*_1,8_(*k*)]*^T^* were modeled according to a dynamic system that fulfills *Eqs. 3** and **4*.

(*3*) g(k)=g(k−1)+εg(k),

(*4*) $$\text{RR}(k)=L{\left(k\right)}^{T}\;g(k)+{\text{ε}}_{\text{RR}}(k),$$

where ε_g_ is the state noise and ε_RR_ is the observation noise. The coefficients were then estimated using a Kalman filter with a time-varying observation matrix (51), and SAI and PAI were estimated as shown in *Eqs. 5* and *6*.

(*5*) $$\text{SAI}\;(k)=[{\text{Ψs}}_{0}+\sum_{j\;=\;1}^{2}{\text{Ψs}}_{j}\;\cdot \;g_{1,\;j-1}(k)]\;/\;\text{RR}(k),$$

(*6*) $$\text{PAI}\;(k)=[{\text{Ψp}}_{0}+\sum_{j\;=\;1}^{7}{\text{Ψp}}_{j}\cdot g_{1,\;j+1}(k)]\cdot 2\text{RR}(k).$$

Here, Ψs*_j_* and Ψp*_j_* are the generalized values for the sympathetic and parasympathetic kernels with numeric values of Ψs*_j_* = {39.2343, 10.1963, −5.9242} and Ψp*_j_* = {28.4875, −17.3627, 5.8798, 12.0628, 5.6408, −7.0664, −5.6779, −3.9474}. For a comprehensive description of the model generation and parametrization, see Ref. (48). SAI and PAI were computed using publicly available online software, which can be gathered from www.saipai-hrv.com.

The validation of SAI and PAI computation has been performed in different studies, including congestive heart failure (52), muscle sympathetic nerve stimulation (53), lower body negative pressure (51), pre-ejection period measurement (54), and controlled breathing (55).

### Functional Brain-Heart Interplay Assessment

The sympathovagal synthetic data generation model (SV-SDG) provides time-variant estimates of the bidirectional functional coupling between heartbeat and brain components. The model uses the estimation of sympathetic and parasympathetic activities proposed in Refs. 42 and 48, described in *Estimation of Sympathetic and Parasympathetic Activities*.

#### Functional interplay from the brain to the heart.

The topdown functional interplay was quantified through a model of synthetic heartbeat generation based on Laguerre expansions of RR series [see Candia-Rivera et al. (42) for further details]. Briefly, heartbeat generation was based on the modulation function *m*(*t*), which contains the fluctuations with respect to the baseline heart rate. Such fluctuations were modeled including the sympathetic and parasympathetic interplay. The modulation function *m*(*t*) was expressed as a linear combination of sympathetic (SAI) and parasympathetic activity index (PAI), and their respective control coefficients *C*_SAI_ and *C*_PAI_, representing the proportional central nervous system contribution:

(*7*) $$m\left(t\right)=C_{\text{SAI}}\left(t\right)\times \text{SAI}\left(t\right)+{\text{C}}_{\text{PAI}}\left(t\right)\times \text{PAI}\left(t\right).$$

The modulation function was then taken as input to an integrate-and-fire model (42). The model was fitted on the RR interval series using a 15-s sliding time window and a linear regression model with no constant term.

*C*_SAI_ and *C*_PAI_ coefficients model the directional interaction from EEG activity to the sympathetic and parasympathetic autonomic components, respectively. Accordingly, the directional interaction from cortical oscillations in each band to the autonomic component modulating heartbeat dynamics was defined as:

(*8*) $${\text{SDG}}_{\text{EEG }F\to X}\left(t\right)=C_{X}\left(t\right)/{\text{EEG}}_{F}\left(t-\text{1}\right),$$

where *X* ∈ {SAI, PAI} and EEG*_F_* indicates the time-varying EEG power with *F* ∈ {δ, θ, α, β, γ}.

#### Functional interplay from the heart to the brain.

The functional interplay from the heart to the brain was quantified through a model based on the generation of synthetic EEG series using an adaptative Markov process (56). The model was fitted using a least-square autoregressive process to estimate cardiac sympathovagal contributions to the ongoing fluctuations in EEG power as:

(*9*) $${\text{EEG}}_{F}\left(t\right)=\kappa_{F}\times {\text{EEG}}_{F}\left(t-\text{1}\right)+\Psi_{F}\left(t-\text{1}\right)+\varepsilon_{F},$$

where *F* is the EEG frequency band, *K_F_* is a fitting constant, ϵ*_F_* is the adjusted error, and Ψ*_F_* indicates the fluctuations of EEG power in *F*. Then, the heart-to-brain functional coupling coefficients were calculated as follows:

(*10*) $${\text{SDG}}_{X}\to_{{\text{EEG}}_{F}}\left(t\right)=\Psi_{F}\left(t\right)/X\left(t\right),$$

where *X* ∈ {SAI, PAI}. For further details, please see Candia-Rivera et al. (38).

The software for the computation of SAI and PAI is available at www.saipai-hrv.com. The source code implementing the SV-SDG model is available at www.github.com/diegocandiar/brain_heart_svsdg.

### Multivariate Analysis

To identify the most significant brain-heart features sensitive to mental stress, a multivariate analysis was performed. The feature selection is based on the ranking provided by the computation of minimum redundancy maximum relevance (MRMR) scores (57) and was computed over the 180 SV-SDG-derived features (180 = 2 directions × 2 autonomic markers × 5 brain oscillations × 9 channels) to select the five most significant ones in two conditions: *1*) a linear regression model predicting the median stress level in each condition, and *2*) a binary classification algorithm to discern low versus high-stress level.

The MRMR score computation algorithm was performed as follows:

1)  The relevance *V_x_* of all features *x* was computed. The feature with the largest relevance $\underset{x\in \Omega }{\text{max}}V_{x}$ was selected. The selected feature was added to an empty set of features *S*.

*V_x_* was defined as:

(*11*) $$V_{x}=\frac{1}{\left|S\right|}\;\sum_{x\;\in S}I(x,\;y)\;,$$

where |S| is the number of features in *S* and *I*(*x, y*) is the mutual information between the feature *x* and the output *y*:

(*12*) $$I\left(x,y\right)=\sum_{ij}p\left(x_{i},y_{j}\right)\text{log}\frac{p\left(x_{i},y_{j}\right)}{p\left(x_{i}\right)p\left(y_{j}\right)}.$$

2)  Next, the features with nonzero relevance *V_x_* and zero redundancy *W_x_* in *S^c^* (complement of *S*) were identified. Then, the feature with the largest relevance was selected, $\underset{x\in S^{c},W_{x}=0}{\text{max}}V_{x}$. The selected feature was added to the set *S*.

*W_x_* was defined as:

(*13*) $$W_{x}=\frac{1}{|S{|}^{2}}\;\sum_{x,z\;\in S}I(x,\;z)\;.$$

If *S^c^* did not include a feature with nonzero relevance and zero redundancy, skip *step 3*

3)  *Step 2* was repeated until the redundancy *W_x_* was not zero for all features in *S^c^*.
4)  The feature with the largest Mutual Information Quotient (MIQ) was selected, with nonzero relevance and nonzero redundancy in *S^c^*, and the selected feature was added to the set *S*.

MIQ was defined as:

(*14*) $$\underset{x\;\in \;S^{c}}{\max}\text{MIQ}=\underset{x\;\in \;S^{c}}{\max}\frac{V_{x}}{W_{x}}=\underset{x\;\in \;S^{c}}{\max}\frac{I(x,y)}{\frac{1}{\left|S\right|}\;\sum_{z\in S}I(x,z)}\;.$$

*5*) *Step 4* was repeated until the relevance was zero for all features in *S^c^*.

*6*) The features with zero relevance were added to *S* in random order.

The multivariate analyses were performed in a fivefold cross-validation framework. Linear regressions to the stress level were performed using least squares kernel regression with regularization strength set to 0.027. The stress level was quantified “0” at rest, “1” for *stressor 1*, “4” for *stressor 2*, and “5” for *stressor 3* to closely match the median stress ratings from subjects’ self-assessment reports. The regression performance was measured through root mean squared error (RMSE) for the prediction of median stress ratings. Binary classification for the low versus high stress recognition was performed through a kernel naïve Bayes classifier with a Gaussian kernel, with “low stress” class associated with “rest” and “*stressor 1*” conditions, and “high stress” associated with the *stressors 2* and *3.* The classification performance was quantified through the classification accuracy.

### Statistical Analysis

Group-wise statistical analysis between the resting state and the three stressor levels was performed through nonparametric Friedman tests, whereas two-condition comparisons were performed through Wilcoxon signed-rank test. The statistical testing was performed per EEG channel, in which the inputs correspond to SV-SDG coupling coefficient computed at different experimental conditions. The significance level of the *P* values was corrected in accordance with the Bonferroni rule for 9 channels, with an uncorrected statistical significance set to α = 0.05. The samples were described group-wise using the median and related dispersion (variability) measures that was quantified though the median absolute deviation (MAD).

## RESULTS

**Figure 2.**
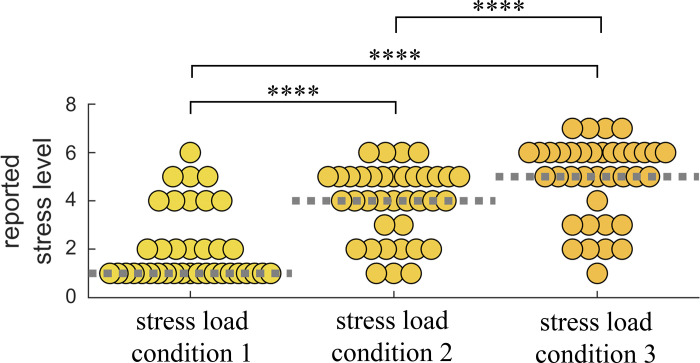
Self-reported stress level for three stressful conditions. Each data point corresponds to the reported stress level per subject for each for: *1*) stress load *condition 1*: documentary, *2*) stress load *condition 2*: documentary + digit span task, *3*) stress load *condition 3*: documentary + digit span task + red box task. ****P < 0.00005 from Wilcoxon signed-rank test.

The participants’ self-reports on the perceived level of stress are displayed in Fig. 2 for each stressful condition, where the group median ± MAD reported stress levels are 1 ± 0, 4 ± 1 and 5 ± 1 (*P* = 2 × 10^−14^ from Friedman test). A multiple comparison analysis showed that the three stressful conditions are significantly different (*P* < 0.00005).

**Figure 3.**
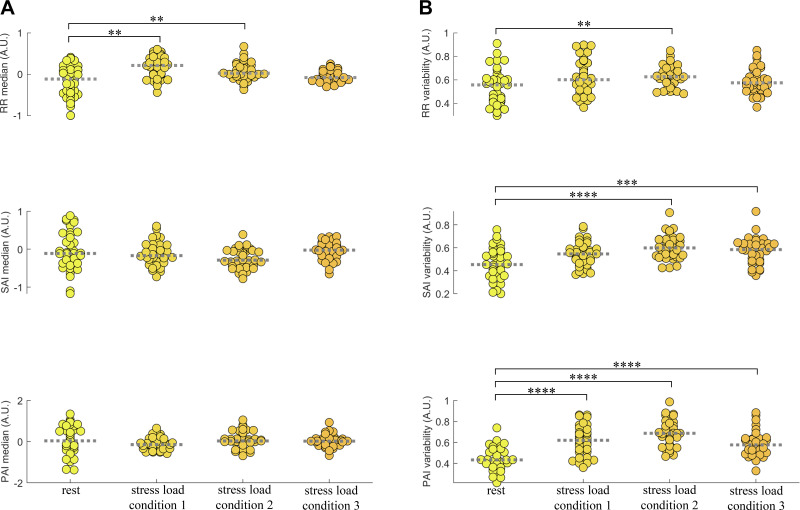
Group-wise distributions of RR, SAI, and PAI median and variability for each experimental condition. Each data point corresponds to the measured autonomic marker per subject at each of the four conditions. *A*: RR, SAI, and PAI median. *B*: RR, SAI, and PAI variability as measured through median absolute deviation (MAD). Time-varying autonomic indexes were z-score normalized for the whole experimental protocol duration before computing median and MAD values. **P < 0.005, ***P < 0.0005, ****P < 0.00005 (Bonferroni-corrected significance at α < 0.00833). PAI, parasympathetic activity index; RR, R-to-R peak interval; SAI, sympathetic activity index.

The cardiac autonomic activity was assessed through the sympathetic and parasympathetic activity indices (SAI and PAI, respectively). Although condensing the SAI and PAI time-resolved information, median SAI and median PAI did not change significantly across the experimental conditions (*P* = 0.0935 from Friedman test on median SAI, and *P* = 0.3101 from Friedman test on median PAI). Nevertheless, SAI and PAI variability (i.e., MAD over time) significantly changes across the experimental conditions (*P* = 7 × 10^−6^ from Friedman test on SAI variability and *P* = 4 × 10^−9^ from Friedman test on PAI variability). Figure 3 depicts group-wise distributions for RR, SAI, and PAI median and variability, with an evident increase in SAI and PAI variability in the three stressful conditions as compared with rest.

**Figure 4.**
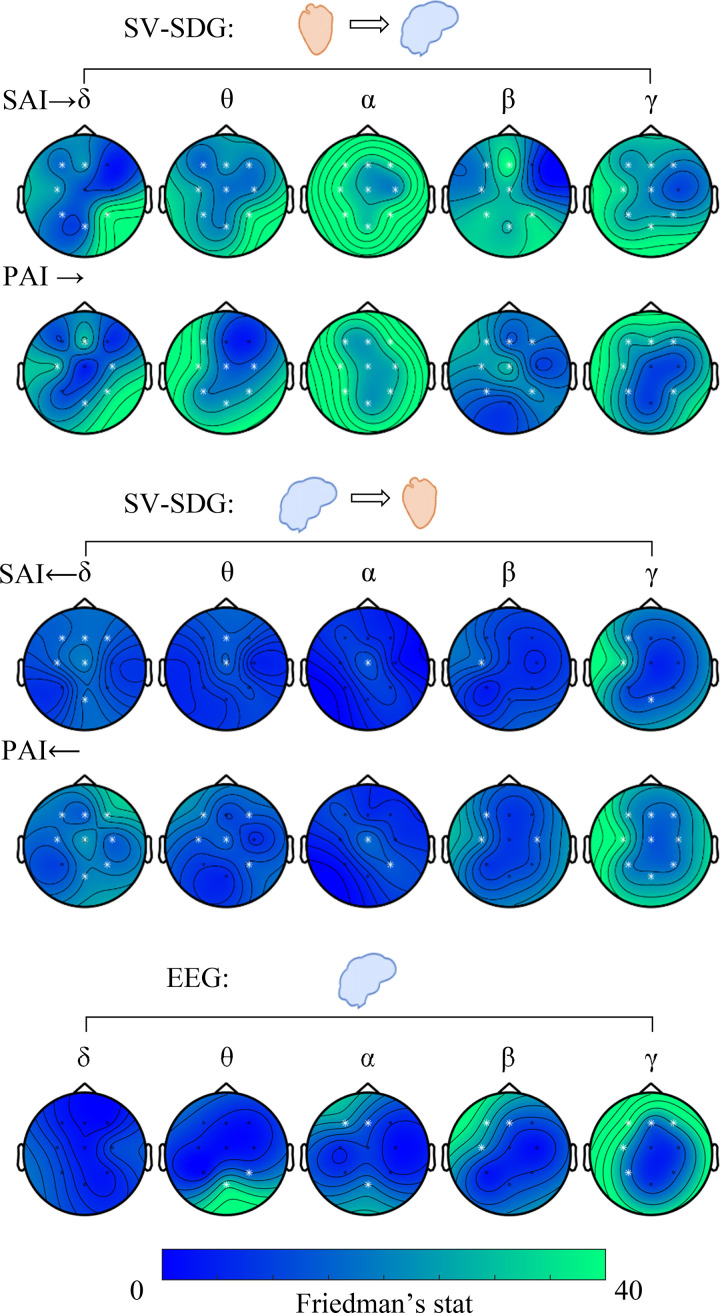
Topographic maps of Friedman nonparametric test for the paired brain-heart interplay variability on the ascending (*top*) and descending (*middle*) direction from the SV-SDG model among experimental conditions (rest and three mental stress conditions). In the *bottom*, topographic maps of Friedman nonparametric test for paired EEG power variability among experimental conditions (rest and three mental stress conditions) are shown. White electrodes indicate *P* < 0.0056. The variability measure was the median absolute deviation. Complementary results on SV-SDG and EEG power medians are in Supplemental Fig. S1. EEG, electroencephalogram; PAI, parasympathetic activity index; SAI, sympathetic activity index; SV-SDG, sympathovagal synthetic data generation.

As autonomic variability is sensitive to stress levels, we further explored how they relate to brain-heart interplay. Figure 4 illustrates results from the Friedman tests on group-wise brain-heart variability changes among experimental conditions. Most of the significant changes among conditions are associated with ascending interactions, especially originating from sympathetic and vagal activity targeting EEG oscillations in the α band (SAI → α: all channels Friedman test *P* ≤ 0.00013, PAI → α: all channels Friedman test *P* ≤ 0.00003). Ascending heart-to-brain communication targeting EEG oscillations in the θ, β, and γ bands show significant changes as well, together with descending interactions from cortical γ oscillations to vagal activity (see Table 1). In contrast, cortical power variability mostly shows not significant changes, with a few statistical differences associated with γ oscillations in the left-frontal electrodes, as shown in Table 2 (see Supplemental Fig. S2 for visualizing an exemplary subject’s SAI, PAI, and γ power fluctuations on time).

**Table 1.** Friedman test results on the variability of brain-heart interplay coupling coefficients

Ascending Coupling Coefficients	Median *P* Value (among Significant Channels)	Median Friedman’s Stat (among Significant Channels)	Significant Channels	Descending Coupling Coefficients	Median *P* Value (among Significant Channels)	Median Friedman’s Stat (among Significant Channels)	Significant Channels
SAI → δ	0.0006	19.5789	F3, Fz, C3, P3, POz, P4	δ → SAI	0.0013	15.7895	F3, Fz, F4, C3, Cz, POz
SAI → θ	0.0001	20.7789	F3, Fz, F4, C3, Cz, C4, P3, POz, P4	θ → SAI	0.0030	13.9579	Fz, Cz
SAI → α	<0.0001	33.8211	F3, Fz, F4, C3, Cz, C4, P3, POz, P4	α → SAI	0.0046	13.0105	Cz
SAI → β	<0.0001	28.7053	F3, Fz, C3, Cz, P3, POz, P4	β → SAI	0.0019	14.9368	C3
SAI → γ	<0.0001	24.0632	F3, Fz, F4, C3, Cz, P3, POz, P4	γ → SAI	0.0004	18	F3, C3, POz
PAI → δ	<0.0001	28.9263	Fz, C3, C4, POz, P4	δ → PAI	0.0006	17.5737	F3, Fz, F4, C3, Cz, C4, POz, P4
PAI → θ	<0.0001	23.6211	F3, C3, Cz, C4, P3, POz, P4	θ → PAI	0.0033	13.7368	F3, F4, C3, Cz, P4
PAI → α	<0.0001	27.6947	F3, Fz, F4, C3, Cz, C4, P3, POz, P4	α → PAI	0.0022	14.9368	Cz, P4
PAI → β	<0.0001	21.6947	F3, Fz, F4, C3, Cz, P3, P4	β → PAI	0.0025	14.3053	F3, C3, C4
PAI → γ	<0.0001	24.4421	F3, Fz, F4, C3 P3, P4	γ → PAI	0.0003	18.7579	F3, Fz, F4, C3, Cz, C4, P3, POz, P4

Median *P* values and *Z* values among significant channels are displayed. Critical α was set according to the Bonferroni rule for multiple comparisons among channels at α = 0.05/9 ≈ 0.0056. Variability of coupling coefficients on time was computed with median absolute deviation (MAD). PAI, parasympathetic activity index; SAI, sympathetic activity index.

**Table 2.** Friedman test results on the variability of EEG power

EEG Power	Median *P* Value (Among Significant Channels)	Median Friedman’s Stat (Among Significant Channels)	Significant Channels
δ	NS	NS	NS
θ	0.0021	22.8158	POz, P4
α	0.0003	18.7895	F3, Fz, POz
β	<0.0001	21.7579	F3, Fz, C3
γ	<0.0001	26.8105	F3, Fz, F4, C3, P3

Median *P* values and *Z* values among significant channels are displayed. Critical α was set according to the Bonferroni rule for multiple comparisons among channels at α = 0.05/9 ≈ 0.0056. Variability of coupling coefficients on time was computed with median absolute deviation (MAD). EEG, electroencephalogram; NS, no significant results.

For the sake of completeness, results on the median brain-heart are shown in Supplemental Fig. S1. Mental stress mainly modulates heart-to-brain functional communication, especially targeting δ, α, β (in the left hemisphere), and γ bands.

**Figure 5.**
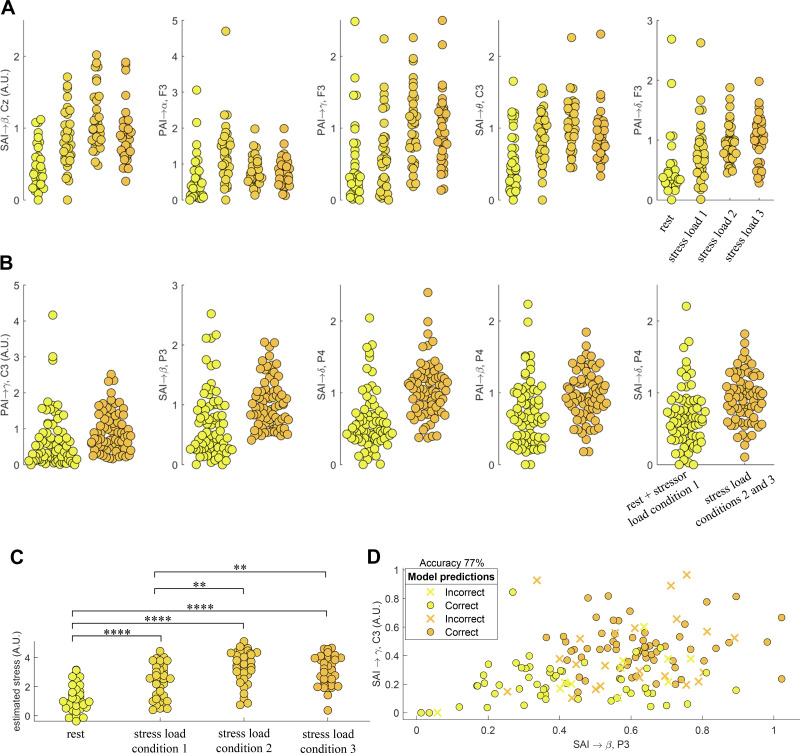
Multivariate analysis results. *A*: the five most informative brain-heart interplay features according to the MRMR regression criteria. The regression was performed on the group median stress level: rest = 0, stressor *condition* 1 = 1, stressor *condition* 2 = 4, stressor *condition* 3 = 5. *B*: the five most informative brain-heart interplay features according to the MRMR criteria to classify low vs. high-stress levels. Low stress referred to rest and stress load *condition 1*; high stress referred to stress load *conditions 2* and *3*. *C*: regression model output from a fivefold cross-validation to the stress level using the best five markers presented in *A*. The statistical comparisons correspond to paired Wilcoxon tests on the estimated stress level score computed with the regression model. *D*: binary stress level classification using the five most informative features in *B*. Yellow and orange circles indicate low and high-stress conditions, respectively. **P < 0.005, ***P < 0.0005, ****P < 0.00005 (Bonferroni-corrected significance at α < 0.00833). MRMR, minimum redundancy maximum relevance.

According to the MRMR algorithm, the five most informative features for the linear regression analysis and the low versus high-stress classification are reported in Table 3 and depicted in Fig. 5. In both multivariate analyses, ascending features from SAI and PAI are prevalent. To illustrate, although median stress level prediction mostly uses SAI → β, most of the information needed for low versus high-stress classification was provided by PAI → γ.

**Table 3.** Brain-heart interplay feature ranking according to the minimum redundancy maximum relevance (MRMR) algorithm

	1st Feature	2nd Feature	3rd Feature	4th Feature	5th Feature
Regression	SAI → β, Cz	PAI → α, F3	PAI → γ, F3	SAI → θ, C3	PAI → δ, P4
	MRMR = 0.2395	MRMR = 0.2247	MRMR = 0.1771	MRMR = 0.1669	MRMR = 0.1521
Binary classification	PAI → γ, C3	SAI → β, P3	SAI → δ, P4	PAI → β, P4	SAI → δ, F3
	MRMR = 0.1464	MRMR = 0.1399	MRMR = 0.1156	MRMR = 0.0473	MRMR = 0.0453

The MRMR scores were computed for two models: regression to the group-median reported stress (rest = 0, stress *condition 1* = 1, stress *condition 2* = 4, stress *condition 3* = 5), and classification of low and high-stress levels (low = rest and stress *condition 1*, high = stress *conditions 2* and *3*). PAI, parasympathetic activity index; SAI, sympathetic activity index.

In the regression analysis, the RMSE was 1.6851, and its output showed a significant difference between all predicted stress levels but *stressor 2* versus *stressor 3* (Fig. 5*C*). In the classification, the five brain-heart features achieved a discrimination accuracy as high as 77% (Fig. 5*D*), with a sensitivity of 85.14% on detecting high stress, and 68.92% specificity.

**Figure 6.**
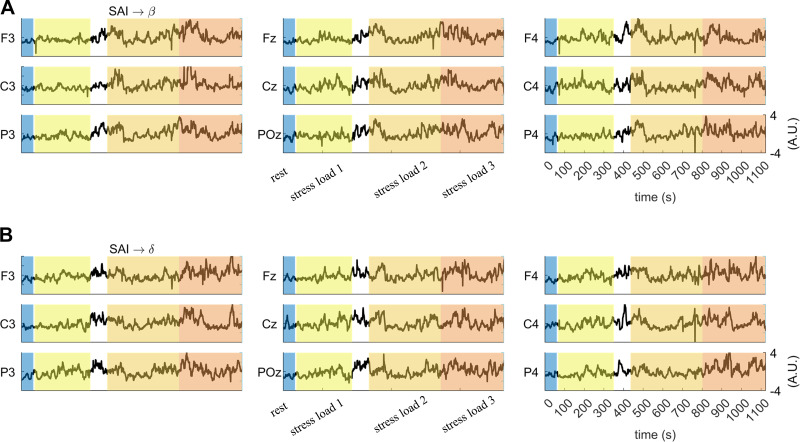
Exemplary participant during the experimental protocol. Parallel fluctuations in (*A*) SAI → β and (*B*) SAI → δ are displayed. SAI, sympathetic activity index.

Figure 6 shows exemplary SAI → β and SAI → δ estimates from one subject for the whole duration of the experimental protocol. An overall increased variability of both markers can be observed in stressful *conditions 2* and *3* with respect to rest and stressful *condition 1*.

## DISCUSSION

Supported by previous evidence linking mental stress with sympathetic activity (15–17) and emotional responses, we investigated functional brain-heart interplay directionality under the hypothesis of modulation across different stress levels.

When condensing the temporal dynamics of sympathetic and parasympathetic activities throughout the experimental conditions, on the one hand, we observed that SAI and PAI central tendencies (median) did not change among stress levels. On the other hand, we observed that the variability (MAD) of SAI and PAI significantly increased in accordance with stress levels up to stressful *condition 2*. Sympathetic activity, as measured through systolic blood pressure, heart rate, ventricular ejection fraction, and skin conductance has been associated with mental stress (22); moreover, mental stress induced by mental arithmetic increases heart rate variability power in the low frequency and a decrease in its high-frequency power (58–61), suggesting an increase in the sympathetic tone, and a decrease in the parasympathetic one. Stress also modulates heartbeat nonlinear dynamics (20, 62). Changes in attention have been referred to as a source of autonomic variability (63). Furthermore, some studies have suggested that high-frequency fluctuations in heartbeat dynamics are associated with memory retrieval, reaction time, and action execution (59, 64, 65), suggesting a dynamic interaction between sympathetic and parasympathetic activities under stress elicitation. As stress elicitation may involve some executive functions (e.g., self-control and working memory), the role of high-frequency autonomic activity has been associated with specific dimensions of executive functioning (66–69).

We observed differences among stressful conditions in EEG oscillations in the γ band. The existing evidence on EEG and stress shows heterogeneous and divergent findings with respect to frequency bands. To illustrate, some studies suggest that different dimensions of stress are associated with α -β interactions (11, 60, 70), θ-β interactions (70, 71), α-γ interactions (72–74), and θ-α interactions (73). Such heterogeneity may be related to the subjectivity and thus high intersubject variability on perceived stress (72), as well as to the coping strategies (75). For instance, the processing of concurrent inputs/tasks requires multiple access to the working memory (76). Another source of variability may be associated with the level of cognitive demand of the tasks, which may not be directly related to the stress level (77). In this study, the digit span task involved a verbal report, which is also associated with EEG signatures of parieto-occipital desynchronizations of lower α (78).

We observed EEG activity modulation as linked to both autonomic branches, whose activity was measured through SAI and PAI. Particularly, our results on the brain-heart interplay show that the variability of ascending heart-to-brain communication reflects the level of stress, especially until stressful *condition 2* as compared with descending brain-to-heart modulations. Indeed, the highest stress level is not statistically associated with the highest variability of heart-to-brain modulation, nor with SAI and PAI dynamics. We speculate this may be due to the following main factors: *1*) perceived stress level is mitigated or masked by mental fatigue due to sustained attention (79) and *2*) the increasing stress conditions may be subject to an attentional-bradycardic effect to hyperarousing conditions (80, 81), also known as “freezing” effect, and thus highest stress conditions may be associated with a different physiological response than other stressful conditions.

Previous studies on physiological correlates of stress focused on top-down mechanisms exclusively (7, 82). Although brain responses may precede cardiac responses, as measured through EEG (60, 74) and fMRI (83, 84), stressors may elicit activity in the amygdala and hippocampus such that a subsequent bottom-up control is activated (85). Indeed, the brain and heart continuously influence each other (84), and the ascending arousal system shapes brain dynamics to mediate awareness of mental states (86), as well as to facilitate performance at different tasks (87, 88) and to shape physical and emotional arousal (37, 38). Stress regulation shares mechanisms involved in emotion regulation as well (34). To illustrate, the anterior insula integrates interoceptive signals during emotional and cognitive processing, being these processes involved in the monitoring of the physiological state of the body (89). The neural monitoring of cardiac inputs may trigger physiological adjustments in the frame of homeostatic and allostatic regulations under emotion elicitation (36, 90). The functional brain-heart interplay under stress elicitation has been shown in heartbeat-evoked potentials correlating with stress-induced changes in cardiac output (22) and correlates of functional connectivity with heart rate variability (31). The role of cardiac inputs in the neurophysiology of stress is also supported by the experimental evidence showing an increased information flow from the heart to the brain during increased attention (63) and disrupted abilities on detecting cardiac and respiratory signals from oneself under anxiety (91, 92).

On the bottom-up modulation, we observed that both sympathetic and vagal oscillations map onto various EEG oscillations at different frequency bands. Indeed, the sympathetic origin of brain-heart interplay in stress was expected because of previous evidence (18, 19, 22, 23). In this study, SAI → β interplay seems more sensitive to changes in stress levels. The involvement of β waves in mental stress has been previously reported (93), along with α-β interactions (11, 60, 70) and θ-β interactions (70, 71). Note that EEG oscillations in the θ band have been consistently reported as a sensitive correlate of emotion processing (94) and also in heart-to-brain communication (38). Our results show that preferential heart-to-brain communication occurs over the frontal and parietal cortical regions, consistently with a previous report on stress (6) and correlates of cognitive operations (95).

We showed that a multivariate analysis helps to distinguish between stress levels, as compared with individual autonomic markers. Although the use of low-density EEG in this study is certainly a limitation to understanding the brain mapping and cortical dynamics of stress neurophysiology, it proves the suitability of this kind of device to detect levels of stress with potential commercial applications. The study of mental stress elicited in other paradigms, such as mental arithmetic, could give a broader view of the physiological processes involved in the brain-heart information exchange. Our study confirms the advantages of analyzing the interactions between the brain and heart, instead of studying heart rate and brain dynamics exclusively (96, 97). The understanding of brain-heart dynamics and the neurophysiological substrate of stress has clinical relevance. Heart rate variability markers are acknowledged to reflect autonomic dysregulation, which may lead to morbidity and mortality (98, 99). The evidence also shows differences in heart rate variability between healthy humans and different mood disorders, but also as a marker of the effects of antidepressant medications (98). The description of stress mechanisms can enlighten the apparent relationships with cardiac death (100), cardiovascular disease (101), sudden death (102), and psychiatric disorders (103). The evidence in other markers of brain-heart interplay shows as well that the dynamic interaction of these systems may relate to different aspects of mental health (22, 104, 105).

This study comes with limitations. The self-assessment measurements we used for subjective stress evaluation do not refer to a specific psychometric test. Moreover, we are aware that confounding factors and artifact sources are numerous in EEG and therefore in functional brain-heart interplay studies (106–108). In particular, the γ activity is quite sensitive to muscle artifacts. Since simultaneously electromyography recordings were not available, we cannot exclude that all of the artifacts have been rejected in our preprocessing stage. This indeed constitutes a limitation of our study. However, by also relying on the several preprocessing steps and by checking all series by visual inspection, we are confident that our results are reliable in highlighting a functional, directional link between EEG oscillations in the γ band and sympathovagal oscillations.

## PERSPECTIVES AND SIGNIFICANCE

Stress neurophysiology involves bidirectional interactions between the brain and heart, with peripheral bodily feedback playing a key role. Mental stress leads to increased variability in sympathetic and parasympathetic activities, which is also reflected in changes in EEG γ activity. These results are in line with the experimental evidence showing a dynamic information exchange between the central and autonomic nervous systems during emotional arousal and physical stress. Estimates of functional brain-heart interplay may be suitable biomarkers of mental stress.

## SUPPLEMENTARY DATA

10.1101/2022.09.09.507362 Supplemental Figs. S1 and S2: https://doi.org/10.1101/2022.09.09.507362.

## GRANTS

The research leading to these results was partially supported by the Italian Ministero dell'Istruzione, dell’Università e della Ricerca (MIUR) in the framework of the FoReLab project (Departments of Excellence) and has received partial funding from the European Commission—Horizon 2020 Program under grant agreement number 813234 (to D. Candia-Rivera and G. Valenza) of the project “RHUMBO” and under grant agreement number 101017727 of the project "EXPERIENCE" (to D. Candia-Rivera and G. Valenza).

## DISCLOSURES

No conflicts of interest, financial or otherwise, are declared by the authors.

## AUTHOR CONTRIBUTIONS

D.C.-R. and T.Z.R. conceived and designed research; K.N. performed experiments; D.C.-R. and G.V. analyzed data; D.C.-R. and G.V. interpreted results of experiments; D.C.-R. prepared figures; D.C.-R. drafted manuscript; D.C.-R., K.N., T.Z.R., and G.V. edited and revised manuscript; D.C.-R., K.N., T.Z.R., and G.V. approved final version of manuscript.

## DATA AVAILABILITY

Data will be made available on reasonable request.

## References

[1] Öhman A, Wiens S. On the automaticity of autonomic responses in emotion: An evolutionary perspective. In: Handbook of Affective Sciences, edited by Davidson RJ, Scherer KR, Goldsmith HH. New York, NY: Oxford University Press, 2003, p. 256–275.

[2] Smith R, Thayer JF, Khalsa SS, Lane RD. The hierarchical basis of neurovisceral integration. Neurosci Biobehav Rev 75: 274–296, 2017. doi:10.1016/j.neubiorev.2017.02.003. 28188890

[3] Gunnar M, Quevedo K. The neurobiology of stress and development. Annu Rev Psychol 58: 145–173, 2007. doi:10.1146/annurev.psych.58.110405.085605. 16903808

[4] Lamotte G, Shouman K, Benarroch EE. Stress and central autonomic network. Auton Neurosci 235: 102870, 2021. doi:10.1016/j.autneu.2021.102870. 34461325

[5] Godoy LD, Rossignoli MT, Delfino-Pereira P, Garcia-Cairasco N, de Lima Umeoka EH. A comprehensive overview on stress neurobiology: basic concepts and clinical implications. Front Behav Neurosci 12: 127, 2018. doi:10.3389/fnbeh.2018.00127. 30034327 PMC6043787

[6] Ridderinkhof KR, Ullsperger M, Crone EA, Nieuwenhuis S. The role of the medial frontal cortex in cognitive control. Science 306: 443–447, 2004. doi:10.1126/science.1100301. 15486290

[7] Janak PH, Tye KM. From circuits to behaviour in the amygdala. Nature 517: 284–292, 2015. doi:10.1038/nature14188. 25592533 PMC4565157

[8] Burgos-Robles A, Kimchi EY, Izadmehr EM, Porzenheim MJ, Ramos-Guasp WA, Nieh EH, Felix-Ortiz AC, Namburi P, Leppla CA, Presbrey KN, Anandalingam KK, Pagan-Rivera PA, Anahtar M, Beyeler A, Tye KM. Amygdala inputs to prefrontal cortex guide behavior amid conflicting cues of reward and punishment. Nat Neurosci 20: 824–835, 2017. doi:10.1038/nn.4553. 28436980 PMC5448704

[9] Godsil BP, Kiss JP, Spedding M, Jay TM. The hippocampal–prefrontal pathway: The weak link in psychiatric disorders? Eur Neuropsychopharmacol 23: 1165–1181, 2013. doi:10.1016/j.euroneuro.2012.10.018. 23332457

[10] Katmah R, Al-Shargie F, Tariq U, Babiloni F, Al-Mughairbi F, Al-Nashash H. A review on mental stress assessment methods using EEG signals. Sensors 21: 5043, 2021. doi:10.3390/s21155043. 34372280 PMC8347831

[11] Vanhollebeke G, De Smet S, De Raedt R, Baeken C, van Mierlo P, Vanderhasselt M-A. The neural correlates of psychosocial stress: A systematic review and meta-analysis of spectral analysis EEG studies. Neurobiol Stress 18: 100452, 2022. doi:10.1016/j.ynstr.2022.100452. 35573807 PMC9095895

[12] Silvani A, Calandra-Buonaura G, Dampney RAL, Cortelli P. Brain-heart interactions: physiology and clinical implications. Philos Trans A Math Phys Eng Sci 374: 20150181, 2016. doi:10.1098/rsta.2015.0181. 27044998

[13] Valenza G, Sclocco R, Duggento A, Passamonti L, Napadow V, Barbieri R, Toschi N. The central autonomic network at rest: uncovering functional MRI correlates of time-varying autonomic outflow. NeuroImage 197: 383–390, 2019. doi:10.1016/j.neuroimage.2019.04.075. 31055043

[14] Valenza G, Passamonti L, Duggento A, Toschi N, Barbieri R. Uncovering complex central autonomic networks at rest: a functional magnetic resonance imaging study on complex cardiovascular oscillations. J R Soc Interface 17: 20190878, 2020. doi:10.1098/rsif.2019.0878. 32183642 PMC7115224

[15] Callister R, Suwarno NO, Seals DR. Sympathetic activity is influenced by task difficulty and stress perception during mental challenge in humans. J Physiol 454: 373–387, 1992. doi:10.1113/jphysiol.1992.sp019269. 1474496 PMC1175610

[16] Kopin IJ. Definitions of stress and sympathetic neuronal responses. Ann N Y Acad Sci 771: 19–30, 1995. doi:10.1111/j.1749-6632.1995.tb44667.x. 8597398

[17] Goldstein DS. 2 - Stress-induced activation of the sympathetic nervous system. Baillieres Clin Endocrinol Metab 1: 253–278, 1987. doi:10.1016/S0950-351X(87)80063-0. 3327494

[18] Vrijkotte TGM, van Doornen LJP, de Geus EJC. Effects of work stress on ambulatory blood pressure, heart rate, and heart rate variability. Hypertension 35: 880–886, 2000. doi:10.1161/01.HYP.35.4.880. 10775555

[19] Hjortskov N, Rissén D, Blangsted AK, Fallentin N, Lundberg U, Søgaard K. The effect of mental stress on heart rate variability and blood pressure during computer work. Eur J Appl Physiol 92: 84–89, 2004. doi:10.1007/s00421-004-1055-z. 14991326

[20] Schubert C, Lambertz M, Nelesen RA, Bardwell W, Choi J-B, Dimsdale JE. Effects of stress on heart rate complexity—a comparison between short-term and chronic stress. Biol Psychol 80: 325–332, 2009. doi:10.1016/j.biopsycho.2008.11.005. 19100813 PMC2653595

[21] Kim H-G, Cheon E-J, Bai D-S, Lee YH, Koo B-H. Stress and heart rate variability: a meta-analysis and review of the literature. Psychiatry Investig 15: 235–245, 2018. doi:10.30773/pi.2017.08.17. 29486547 PMC5900369

[22] Gray MA, Taggart P, Sutton PM, Groves D, Holdright DR, Bradbury D, Brull D, Critchley HD. A cortical potential reflecting cardiac function. Proc Natl Acad Sci USA 104: 6818–6823, 2007. doi:10.1073/pnas.0609509104. 17420478 PMC1871868

[23] Greco A, Valenza G, Lazaro J, Garzon-Rey JM, Aguilo J, De-la-Camara C, Bailon R, Scilingo EP. Acute stress state classification based on electrodermal activity modeling. IEEE Trans Affect Comput 14: 788–799, 2021. doi:10.1109/TAFFC.2021.3055294.

[24] Boiten FA, Frijda NH, Wientjes CJE. Emotions and respiratory patterns: review and critical analysis. Int J Psychophysiol 17: 103–128, 1994. doi:10.1016/0167-8760(94)90027-2. 7995774

[25] Vinkers CH, Penning R, Hellhammer J, Verster JC, Klaessens JHGM, Olivier B, Kalkman CJ. The effect of stress on core and peripheral body temperature in humans. Stress 16: 520–530, 2013. doi:10.3109/10253890.2013.807243. 23790072

[26] Nakamura K, Morrison SF. Central sympathetic network for thermoregulatory responses to psychological stress. Auton Neurosci 237: 102918, 2022. doi:10.1016/j.autneu.2021.102918. 34823147

[27] Collins SM. IV. Modulation of intestinal inflammation by stress: basic mechanisms and clinical relevance. Am J Physiol Gastrointest Liver Physiol 280: G315–G318, 2001. doi:10.1152/ajpgi.2001.280.3.G315. 11171612

[28] Söderholm JD, Perdue MH. Stress and gastrointestinal tract. II. Stress and intestinal barrier function. Am J Physiol Gastrointest Liver Physiol 280: G7–G13, 2001. doi:10.1152/ajpgi.2001.280.1.G7. 11123192

[29] Ulrich-Lai YM, Herman JP. Neural regulation of endocrine and autonomic stress responses. Nat Rev Neurosci 10: 397–409, 2009. doi:10.1038/nrn2647. 19469025 PMC4240627

[30] Segerstrom SC, Miller GE. Psychological stress and the human immune system: a meta-analytic study of 30 years of inquiry. Psychol Bull 130: 601–630, 2004. doi:10.1037/0033-2909.130.4.601. 15250815 PMC1361287

[31] Chand T, Li M, Jamalabadi H, Wagner G, Lord A, Alizadeh S, Danyeli LV, Herrmann L, Walter M, Sen ZD. Heart rate variability as an index of differential brain dynamics at rest and after acute stress induction. Front Neurosci 14: 645, 2020. doi:10.3389/fnins.2020.00645. 32714132 PMC7344021

[32] Pernice R, Antonacci Y, Zanetti M, Busacca A, Marinazzo D, Faes L, Nollo G. Multivariate correlation measures reveal structure and strength of brain–body physiological networks at rest and during mental stress. Front Neurosci 14: 602584, 2021. doi:10.3389/fnins.2020.602584. 33613173 PMC7890264

[33] McEwen BS. Physiology and neurobiology of stress and adaptation: central role of the brain. Physiol Rev 87: 873–904, 2007. doi:10.1152/physrev.00041.2006. 17615391

[34] Thayer JF, Mather M, Koenig J. Stress and aging: a neurovisceral integration perspective. Psychophysiology 58: e13804, 2021. doi:10.1111/psyp.13804. 33723899

[35] Kreibig SD. Autonomic nervous system activity in emotion: a review. Biol Psychol 84: 394–421, 2010. doi:10.1016/j.biopsycho.2010.03.010. 20371374

[36] Pace-Schott EF, Amole MC, Aue T, Balconi M, Bylsma LM, Critchley H, Demaree HA, Friedman BH, Gooding AEK, Gosseries O, Jovanovic T, Kirby LAJ, Kozlowska K, Laureys S, Lowe L, Magee K, Marin M-F, Merner AR, Robinson JL, Smith RC, Spangler DP, Van Overveld M, VanElzakker MB. Physiological feelings. Neurosci Biobehav Rev 103: 267–304, 2019. doi:10.1016/j.neubiorev.2019.05.002. 31125635

[37] Candia-Rivera D, Catrambone V, Barbieri R, Valenza G. Functional assessment of bidirectional cortical and peripheral neural control on heartbeat dynamics: a brain-heart study on thermal stress. NeuroImage 251: 119023, 2022. doi:10.1016/j.neuroimage.2022.119023. 35217203

[38] Candia-Rivera D, Catrambone V, Thayer JF, Gentili C, Valenza G. Cardiac sympathetic-vagal activity initiates a functional brain–body response to emotional arousal. Proc Natl Acad Sci USA 119: e2119599119, 2022. doi:10.1073/pnas.2119599119. 35588453 PMC9173754

[39] Füstös J, Gramann K, Herbert BM, Pollatos O. On the embodiment of emotion regulation: interoceptive awareness facilitates reappraisal. Soc Cogn Affect Neurosci 8: 911–917, 2013. doi:10.1093/scan/nss089. 22933520 PMC3831556

[40] Klein AS, Dolensek N, Weiand C, Gogolla N. Fear balance is maintained by bodily feedback to the insular cortex in mice. Science 374: 1010–1015, 2021. doi:10.1126/science.abj8817. 34793231

[41] Schulz A, Vögele C. Interoception and stress. Front Psychol 6: 993, 2015. doi:10.3389/fpsyg.2015.00993. 26257668 PMC4507149

[42] Candia-Rivera D, Catrambone V, Barbieri R, Valenza G. Integral pulse frequency modulation model driven by sympathovagal dynamics: synthetic vs. real heart rate variability. Biomed Signal Process Control 68: 102736, 2021. doi:10.1016/j.bspc.2021.102736.

[43] Northoff G, Schneider F, Rotte M, Matthiae C, Tempelmann C, Wiebking C, Bermpohl F, Heinzel A, Danos P, Heinze H-J, Bogerts B, Walter M, Panksepp J. Differential parametric modulation of self-relatedness and emotions in different brain regions. Hum Brain Mapp 30: 369–382, 2009. doi:10.1002/hbm.20510. 18064583 PMC6870760

[44] Pinel P, Dehaene S, Rivière D, LeBihan D. Modulation of parietal activation by semantic distance in a number comparison task. NeuroImage 14: 1013–1026, 2001. doi:10.1006/nimg.2001.0913. 11697933

[45] Oostenveld R, Fries P, Maris E, Schoffelen J-M. FieldTrip: open source software for advanced analysis of MEG, EEG, and invasive electrophysiological data. Comput Intell Neurosci 9: 156869, 2011. doi:10.1155/2011/156869. 21253357 PMC3021840

[46] Candia-Rivera D, Catrambone V, Valenza G. The role of electroencephalography electrical reference in the assessment of functional brain–heart interplay: from methodology to user guidelines. J Neurosci Methods 360: 109269, 2021. doi:10.1016/j.jneumeth.2021.109269. 34171310

[47] Citi L, Brown EN, Barbieri R. A real-time automated point process method for detection and correction of erroneous and ectopic heartbeats. IEEE Trans Biomed Eng 59: 2828–2837, 2012. doi:10.1109/TBME.2012.2211356. 22875239 PMC3523127

[48] Valenza G, Citi L, Saul JP, Barbieri R. Measures of sympathetic and parasympathetic autonomic outflow from heartbeat dynamics. J Appl Physiol (1985) 125: 19–39, 2018. doi:10.1152/japplphysiol.00842.2017. 29446712

[49] Goldstein DS, Bentho O, Park M-Y, Sharabi Y. Low-frequency power of heart rate variability is not a measure of cardiac sympathetic tone but may be a measure of modulation of cardiac autonomic outflows by baroreflexes. Exp Physiol 96: 1255–1261, 2011. doi:10.1113/expphysiol.2010.056259. 21890520 PMC3224799

[50] Reyes del Paso GA, Langewitz W, Mulder LJM, van Roon A, Duschek S. The utility of low frequency heart rate variability as an index of sympathetic cardiac tone: a review with emphasis on a reanalysis of previous studies. Psychophysiology 50: 477–487, 2013. doi:10.1111/psyp.12027. 23445494

[51] Valenza G, Citi L, Wyller VB, Barbieri R. ECG-derived sympathetic and parasympathetic activity in the healthy: an early lower-body negative pressure study using adaptive Kalman prediction. Annu Int Conf IEEE Eng Med Biol Soc 2018: 5628–5631, 2018. doi:10.1109/EMBC.2018.8513512. 30441612

[52] Valenza G, Citi L, Saul JP, Barbieri R. ECG-derived sympathetic and parasympathetic nervous system dynamics: a congestive heart failure study. In: 2018 Computing in Cardiology Conference (CinC). Maastricht, Netherlands, 2018, p. 1–4. doi:10.22489/CinC.2018.282.

[53] Valenza G, Faita F, Citi L, Saul J, Bruno R, Barbieri R. Validation of sympathetic activity index from heart rate variability series: a preliminary muscle sympathetic nerve activity study. In: 2020 Computing in Cardiology. Rimini, Italy, 2020, p. 1–4. doi:10.22489/CinC.2020.365.

[54] Valenza G, Nackley B, Friedman BH, Barbieri R. Validation of sympathetic activity index from heartbeat series: a preliminary study on pre-ejection periods. In: 2022 12th Conference of the European Study Group on Cardiovascular Oscillations (ESGCO). Vysoké Tatry, Štrbské Pleso, Slovakia, 2022, p. 1–2. doi:10.1109/ESGCO55423.2022.9931355.

[55] Valenza G, Saul JP, Barbieri R. Heart rate variability in spontaneous and controlled breathing: a HF power vs. parasympathetic activity index study. In: 2022 12th Conference of the European Study Group on Cardiovascular Oscillations (ESGCO). Vysoké Tatry, Štrbské Pleso, Slovakia, 2022, p. 1–2. doi:10.1109/ESGCO55423.2022.9931393.

[56] Al-Nashash H, Al-Assaf Y, Paul J, Thakor N. EEG signal modeling using adaptive Markov process amplitude. IEEE Trans Biomed Eng 51: 744–751, 2004. doi:10.1109/TBME.2004.826602. 15132500

[57] Peng H, Long F, Ding C. Feature selection based on mutual information: criteria of max-dependency, max-relevance, and min-redundancy. IEEE Trans Pattern Anal Mach Intell 27: 1226–1238, 2005. doi:10.1109/TPAMI.2005.159. 16119262

[58] Dzhebrailova TD, Korobeinikova II, Karatygin NA, Dudnik EN. Dynamics of EEG α activity and heart rate variability in subjects performing cognitive tests. Hum Physiol 41: 599–610, 2015. doi:10.1134/S0362119715040076. 26859987

[59] Wang X, Liu B, Xie L, Yu X, Li M, Zhang J. Cerebral and neural regulation of cardiovascular activity during mental stress. Biomed Eng Online 15: 160, 2016. doi:10.1186/s12938-016-0255-1. 28155673 PMC5260034

[60] Yu X, Zhang J, Xie D, Wang J, Zhang C. Relationship between scalp potential and autonomic nervous activity during a mental arithmetic task. Auton Neurosci 146: 81–86, 2009. doi:10.1016/j.autneu.2008.12.005. 19171503

[61] Yu X, Zhang J. Estimating the cortex and autonomic nervous activity during a mental arithmetic task. Biomed Signal Process Control 7: 303–308, 2012. doi:10.1016/j.bspc.2011.06.001.

[62] Delliaux S, Delaforge A, Deharo J-C, Chaumet G. Mental workload alters heart rate variability, lowering non-linear dynamics. Front Physiol 10: 565, 2019. doi:10.3389/fphys.2019.00565. 31156454 PMC6528181

[63] Kumar M, Singh D, Deepak KK. Identifying heart-brain interactions during internally and externally operative attention using conditional entropy. Biomed Signal Process Control 57: 101826, 2020. doi:10.1016/j.bspc.2019.101826.

[64] Hansen AL, Johnsen BH, Thayer JF. Vagal influence on working memory and attention. Int J Psychophysiol 48: 263–274, 2003. doi:10.1016/S0167-8760(03)00073-4. 12798986

[65] Salvia E, Guillot A, Collet C. The effects of mental arithmetic strain on behavioral and physiological responses. J Psychophysiol 27: 173–184, 2013. doi:10.1027/0269-8803/a000102.

[66] Duschek S, Muckenthaler M, Werner N, Reyes del Paso GA. Relationships between features of autonomic cardiovascular control and cognitive performance. Biol Psychol 81: 110–117, 2009. doi:10.1016/j.biopsycho.2009.03.003. 19428975

[67] Thayer JF, Hansen AL, Saus-Rose E, Johnsen BH. Heart rate variability, prefrontal neural function, and cognitive performance: the neurovisceral integration perspective on self-regulation, adaptation, and health. Ann Behav Med 37: 141–153, 2009. doi:10.1007/s12160-009-9101-z. 19424767

[68] Williams PG, Cribbet MR, Tinajero R, Rau HK, Thayer JF, Suchy Y. The association between individual differences in executive functioning and resting high-frequency heart rate variability. Biol Psychol 148: 107772, 2019. doi:10.1016/j.biopsycho.2019.107772. 31577925

[69] Magnon V, Vallet GT, Benson A, Mermillod M, Chausse P, Lacroix A, Bouillon-Minois J-B, Dutheil F. Does heart rate variability predict better executive functioning? A systematic review and meta-analysis. Cortex 155: 218–236, 2022. doi:10.1016/j.cortex.2022.07.008. 36030561

[70] Wen TY, Aris SAM. Electroencephalogram (EEG) stress analysis on alpha/beta ratio and theta/beta ratio. Int J Environ Res Public Health 17: 175–182, 2020. doi:10.11591/ijeecs.v17.i1.pp175-182. 34886531

[71] Putman P, Verkuil B, Arias-Garcia E, Pantazi I, van Schie C. EEG theta/beta ratio as a potential biomarker for attentional control and resilience against deleterious effects of stress on attention. Cogn Affect Behav Neurosci 14: 782–791, 2014. doi:10.3758/s13415-013-0238-7. 24379166

[72] Luijcks R, Vossen CJ, Hermens HJ, Os J. V, Lousberg R. The influence of perceived stress on cortical reactivity: a proof-of-principle study. PLoS One 10: e0129220, 2015. doi:10.1371/journal.pone.0129220. 26090882 PMC4475054

[73] Micheloyannis S, Sakkalis V, Vourkas M, Stam CJ, Simos PG. Neural networks involved in mathematical thinking: evidence from linear and non-linear analysis of electroencephalographic activity. Neurosci Lett 373: 212–217, 2005. doi:10.1016/j.neulet.2004.10.005. 15619545

[74] Umeno K, Hori E, Tabuchi E, Takakura H, Miyamoto K, Ono T, Nishijo H. Gamma-band EEGs predict autonomic responses during mental arithmetic. NeuroReport 14: 477–480, 2003. doi:10.1097/00001756-200303030-00036. 12634507

[75] Hinault T, Lemaire P. What does EEG tell us about arithmetic strategies? A review. Int J Psychophysiol 106: 115–126, 2016. doi:10.1016/j.ijpsycho.2016.05.006. 27220781

[76] Kim C, Johnson NF, Gold BT. Conflict adaptation in prefrontal cortex: now you see it, now you don’t. Cortex 50: 76–85, 2014. doi:10.1016/j.cortex.2013.08.011. 24074459 PMC3872513

[77] Fink A, Grabner RH, Neuper C, Neubauer AC. EEG alpha band dissociation with increasing task demands. Brain Res Cogn Brain Res 24: 252–259, 2005. doi:10.1016/j.cogbrainres.2005.02.002. 15993763

[78] Grabner RH, De Smedt B. Neurophysiological evidence for the validity of verbal strategy reports in mental arithmetic. Biol Psychol 87: 128–136, 2011. doi:10.1016/j.biopsycho.2011.02.019. 21382434

[79] Xiao Y, Ma F, Lv Y, Cai G, Teng P, Xu F, Chen S. Sustained attention is associated with error processing impairment: evidence from mental fatigue study in four-choice reaction time task. PLoS One 10: e0117837, 2015. doi:10.1371/journal.pone.0117837. 25756780 PMC4355415

[80] Bradley MM, Codispoti M, Cuthbert BN, Lang PJ. Emotion and motivation I: defensive and appetitive reactions in picture processing. Emotion 1: 276–298, 2001. doi:10.1037/1528-3542.1.3.276. 12934687

[81] Valenza G, Greco A, Gentili C, Lanata A, Sebastiani L, Menicucci D, Gemignani A, Scilingo EP. Combining electroencephalographic activity and instantaneous heart rate for assessing brain–heart dynamics during visual emotional elicitation in healthy subjects. Philos Trans A Math Phys Eng Sci 374: 20150176, 2016. doi:10.1098/rsta.2015.0176. 27044990 PMC4822439

[82] Radley J, Morilak D, Viau V, Campeau S. Chronic stress and brain plasticity: mechanisms underlying adaptive and maladaptive changes and implications for stress-related CNS disorders. Neurosci Biobehav Rev 58: 79–91, 2015. doi:10.1016/j.neubiorev.2015.06.018. 26116544 PMC4684432

[83] Iacovella V, Faes L, Hasson U. Task-induced deactivation in diverse brain systems correlates with interindividual differences in distinct autonomic indices. Neuropsychologia 113: 29–42, 2018. doi:10.1016/j.neuropsychologia.2018.03.005. 29530799

[84] Pfurtscheller G, Blinowska KJ, Kaminski M, Schwerdtfeger AR, Rassler B, Schwarz G, Klimesch W. Processing of fMRI-related anxiety and bi-directional information flow between prefrontal cortex and brain stem. Sci Rep 11: 22348, 2021. doi:10.1038/s41598-021-01710-8. 34785719 PMC8595881

[85] Arnsten AFT. Stress signalling pathways that impair prefrontal cortex structure and function. Nat Rev Neurosci 10: 410–422, 2009. doi:10.1038/nrn2648. 19455173 PMC2907136

[86] Munn BR, Müller EJ, Wainstein G, Shine JM. The ascending arousal system shapes neural dynamics to mediate awareness of cognitive states. Nat Commun 12: 6016, 2021. doi:10.1038/s41467-021-26268-x. 34650039 PMC8516926

[87] Fujimoto A, Murray EA, Rudebeck PH. Interaction between decision-making and interoceptive representations of bodily arousal in frontal cortex. Proc. Natl. Acad. Sci. USA 118: e2014781118, 2021. doi:10.1073/pnas.2014781118. 34452993 PMC8536360

[88] Skora LI, Livermore JJA, Roelofs K. The functional role of cardiac activity in perception and action. Neurosci Biobehav Rev 137: 104655, 2022. doi:10.1016/j.neubiorev.2022.104655. 35395334

[89] Craig AD. How do you feel — now? The anterior insula and human awareness. Nat Rev Neurosci 10: 59–70, 2009. doi:10.1038/nrn2555. 19096369

[90] Barrett LF, Simmons WK. Interoceptive predictions in the brain. Nat Rev Neurosci 16: 419–429, 2015. doi:10.1038/nrn3950. 26016744 PMC4731102

[91] Garfinkel SN, Manassei MF, Hamilton-Fletcher G, In den Bosch Y, Critchley HD, Engels M. Interoceptive dimensions across cardiac and respiratory axes. Philos Trans R Soc Lond B Biol Sci 371: 20160014, 2016. doi:10.1098/rstb.2016.0014. 28080971 PMC5062102

[92] Harrison OK, Köchli L, Marino S, Luechinger R, Hennel F, Brand K, Hess AJ, Frässle S, Iglesias S, Vinckier F, Petzschner FH, Harrison SJ, Stephan KE. Interoception of breathing and its relationship with anxiety. Neuron 109: 4080–4093.e8, 2021. doi:10.1016/j.neuron.2021.09.045. 34672986 PMC8691949

[93] Hayashi T, Okamoto E, Nishimura H, Mizuno-Matsumoto Y, Ishii R, Ukai S. Beta activities in EEG associated with emotional stress. Int J Intell Comput Med Sci Image Process 3: 57–68, 2009. doi:10.1080/1931308X.2009.10644171.

[94] Bekkedal MYV, Rossi J 3rd, Panksepp J. Human brain EEG indices of emotions: Delineating responses to affective vocalizations by measuring frontal theta event-related synchronization. Neurosci Biobehav Rev 35: 1959–1970, 2011. doi:10.1016/j.neubiorev.2011.05.001. 21596060

[95] Gruber O, Indefrey P, Steinmetz H, Kleinschmidt A. Dissociating neural correlates of cognitive components in mental calculation. Cereb Cortex 11: 350–359, 2001. doi:10.1093/cercor/11.4.350. 11278198

[96] Azzalini D, Rebollo I, Tallon-Baudry C. Visceral signals shape brain dynamics and cognition. Trends Cogn Sci 23: 488–509, 2019. doi:10.1016/j.tics.2019.03.007. 31047813

[97] Candia-Rivera D. Brain-heart interactions in the neurobiology of consciousness. Curr Res Neurobiol 3: 100050, 2022. doi:10.1016/j.crneur.2022.100050. 36685762 PMC9846460

[98] Kemp AH, Quintana DS. The relationship between mental and physical health: Insights from the study of heart rate variability. Int J Psychophysiol 89: 288–296, 2013. doi:10.1016/j.ijpsycho.2013.06.018. 23797149

[99] Samuels MA. The brain–heart connection. Circulation 116: 77–84, 2007. doi:10.1161/CIRCULATIONAHA.106.678995. 17606855

[100] Critchley HD, Taggart P, Sutton PM, Holdright DR, Batchvarov V, Hnatkova K, Malik M, Dolan RJ. Mental stress and sudden cardiac death: asymmetric midbrain activity as a linking mechanism. Brain 128: 75–85, 2005. doi:10.1093/brain/awh324. 15496434

[101] Hering D, Lachowska K, Schlaich M. Role of the sympathetic nervous system in stress-mediated cardiovascular disease. Curr Hypertens Rep 17: 80, 2015. doi:10.1007/s11906-015-0594-5. 26318888

[102] La Rovere MT, Gorini A, Schwartz PJ. Stress, the autonomic nervous system, and sudden death. Auton Neurosci 237: 102921, 2022. doi:10.1016/j.autneu.2021.102921. 34823148

[103] Gurel NZ, Hadaya J, Ardell JL. Stress-related dysautonomias and neurocardiology-based treatment approaches. Auton Neurosci 239: 102944, 2022. doi:10.1016/j.autneu.2022.102944. 35158161

[104] Catrambone V, Messerotti Benvenuti S, Gentili C, Valenza G. Intensification of functional neural control on heartbeat dynamics in subclinical depression. Transl Psychiatry 11: 221, 2021. doi:10.1038/s41398-021-01336-4. 33854037 PMC8046790

[105] Iseger TA, Bueren N. V, Kenemans JL, Gevirtz R, Arns M. A frontal-vagal network theory for major depressive disorder: implications for optimizing neuromodulation techniques. Brain Stimul 13: 1–9, 2020. doi:10.1016/j.brs.2019.10.006. 31668983

[106] Buot A, Azzalini D, Chaumon M, Tallon-Baudry C. Does stroke volume influence heartbeat evoked responses? Biol Psychol 165: 108165, 2021. doi:10.1016/j.biopsycho.2021.108165. 34416348

[107] Candia-Rivera D, Sappia MS, Horschig JM, Colier WNJM, Valenza G. Confounding effects of heart rate, breathing rate, and frontal fNIRS on interoception. Sci Rep 12: 20701, 2022. doi:10.1038/s41598-022-25119-z. 36450811 PMC9712694

[108] Muthukumaraswamy S. High-frequency brain activity and muscle artifacts in MEG/EEG: a review and recommendations. Front Hum Neurosci 7: 138, 2013. doi:10.3389/fnhum.2013.00138. 23596409 PMC3625857

